# A review of health utilities across conditions common in paediatric and adult populations

**DOI:** 10.1186/1477-7525-8-12

**Published:** 2010-01-27

**Authors:** Jean-Eric Tarride, Natasha Burke, Matthias Bischof, Robert B Hopkins, Linda Goeree, Kaitryn Campbell, Feng Xie, Daria O'Reilly, Ron Goeree

**Affiliations:** 1 Programs for Assessment of Technology in Health (PATH) Research Institute, St Joseph's Healthcare Hamilton, Ontario, Canada; 2 Department of Clinical Epidemiology & Biostatistics, Faculty of Health Sciences, McMaster University, Hamilton, Ontario, Canada

## Abstract

**Background:**

Cost-utility analyses are commonly used in economic evaluations of interventions or conditions that have an impact on health-related quality of life. However, evaluating utilities in children presents several challenges since young children may not have the cognitive ability to complete measurement tasks and thus utility values must be estimated by proxy assessors. Another solution is to use utilities derived from an adult population. To better inform the future conduct of cost-utility analyses in paediatric populations, we reviewed the published literature reporting utilities among children and adults across selected conditions common to paediatric and adult populations.

**Methods:**

An electronic search of Ovid MEDLINE, EMBASE, and the Cochrane Library up to November 2008 was conducted to identify studies presenting utility values derived from the Health Utilities Index (HUI) or EuroQoL-5Dimensions (EQ-5D) questionnaires or using time trade off (TTO) or standard gamble (SG) techniques in children and/or adult populations from randomized controlled trials, comparative or non-comparative observational studies, or cross-sectional studies. The search was targeted to four chronic diseases/conditions common to both children and adults and known to have a negative impact on health-related quality of life (HRQoL).

**Results:**

After screening 951 citations identified from the literature search, 77 unique studies included in our review evaluated utilities in patients with asthma (n = 25), cancer (n = 23), diabetes mellitus (n = 11), skin diseases (n = 19) or chronic diseases (n = 2), with some studies evaluating multiple conditions. Utility values were estimated using HUI (n = 33), EQ-5D (n = 26), TTO (n = 12), and SG (n = 14), with some studies applying more than one technique to estimate utility values. 21% of studies evaluated utilities in children, of those the majority being in the area of oncology. No utility values for children were reported in skin diseases. Although few studies provided comparative information on utility values between children and adults, results seem to indicate that utilities may be similar in adolescents and young adults with asthma and acne. Differences in results were observed depending on methods and proxies.

**Conclusions:**

This review highlights the need to conduct future research regarding measurement of utilities in children.

## Background

The rising cost of healthcare has led to an increased use of economic evaluations to evaluate the costs and consequences of healthcare interventions (e.g. pharmacotherapies, medical devices). In addition to demonstrating that a new product is safe and effective, economic evaluations are now required in many constituencies to obtain reimbursement. When healthcare interventions have an impact on patients' health-related quality of life (HRQoL), several jurisdictions (e.g. Canada, UK) recommend the use of cost-utility analyses (CUAs) as the reference case [1]. In CUAs, the consequences of the interventions are valued in terms of quality-adjusted life-years (QALYs) where QALYs are a composite measure of outcome where utilities for health states (on 0-1 scale where 0 corresponds to death and 1 to full health) act as qualitative weights to combine quantity with quality of life.

A key aspect in conducting a CUA is to determine the utility or health preference associated with particular health states (e.g., sick). Utilities can be taken from the literature but values from the literature, if available, may not always be relevant to the health states and population of interest. Utilities can also be derived from expert opinion when physicians, nurses or other experts are asked to provide a judgment regarding the utility value for a disease or a range of health states (e.g. well, sick and dead). However, because this method has several limitations (e.g. who's judgment, how obtained, how much experience, how consensus is reached), it is recommended to measure utilities through formal direct or indirect measurements. Direct measurements involve the use of standard gamble (SG) or time trade-off (TTO) techniques to elicit preferences for particular health states. In both cases, scenarios specific to the study are developed and face to face interviews are conducted to observe when the individual is indifferent between a gamble (e.g. live with disease A until death or receive an intervention which can cure or kill you immediately with probability p) or a TTO (live with disease A until death or live a few years less but in a better health state). Indirect measurements of utility refer to the use of pre-developed preferences questionnaires such as the European EuroQoL-5 Dimensions (EQ-5D) or the Canadian Health Utility Index (HUI) self-administered questionnaires. Here, patients (children or adults) or proxies rate their health-related quality of life according to the dimensions included in the instrument (e.g. for example, mobility, self-care, usual activities, pain/discomfort and anxiety/depression for the EQ-5D). Patients' (proxies') ratings are then converted to a health utility score using a scoring algorithm based on the preferences of the general adult public.

Although both direct (i.e. using TTO or SG techniques) and indirect (i.e. using pre-existing questionnaires) measurements are commonly used when performing CUAs in adult populations, collecting utilities in children and adolescents presents several challenges. Young children may not have the cognitive ability to complete measurement tasks and thus proxies (e.g. parents, clinicians) are used to estimate HRQoL or utility values [2]. It may also be difficult in some cases to separate the true effect of a healthcare intervention from the normal development of the children (e.g. autonomy). Preference-based instruments such as the EQ-5D were developed for adult populations and may not include other dimensions relevant to children and adolescents (e.g. body image) [2]. One preference-based instrument which was specifically developed for use in children with cancer is the Health Utilities Index Mark (HUI)-2. Although the Child Health Utility 9D (CHU 9D) instrument was recently developed for use in paediatric economic evaluations [3], studies using this instrument to estimate utility values in children have yet to be published. It should also be noted that even if the HUI-2 or the CHU9D are administered to children, the preferences used to valuate the children' ratings into a utility via a scoring algorithm are derived from the adult general public.

Another alternative to derive utilities for a paediatric population is to elicit preferences from the general public through direct measurements techniques. In this situation, adults are asked to imagine that they are children with a certain disease before being invited to express their preferences for particular health states using SG or TTO techniques. However, this task is both resource intensive (e.g., need to develop health states scenarios) and time intensive (e.g., 20-30 minutes for each individual face-to-face interview) compared to pre-existing questionnaire (3-7 minutes for the EQ-5D or HUI questionnaires). In addition, these measurements may be subject to interpretation (e.g. asking an adult to imagine that he/she is a child with a given disease).

It is therefore not surprising that a review by Griebsch et al. of 53 cost-utility studies in paediatrics (i.e. patients were 16 years of age or younger) published before April 2004 reported that authors' or clinicians' judgment was used in 35% of the studies (n = 23) [4]. A smaller proportion (17%) of studies administered the HUI (n = 12) and the EQ-5D (n = 5) questionnaires while TTO and SG techniques were the method of choice in 11 studies. The remaining studies used other methods (n = 7) or did not state the methods (17% or n = 11). In terms of the source of the preferences, author/clinician and the general public represented 40% and 37% respectively of the sources used to calculate utilities in children under the age of 16 years [4]. In comparison, 10% of the studies used preferences from adult patients, 5% used parents as proxies and only 2% of the studies used children as the source of the preferences. These results should, however, be interpreted with caution as half of these 53 cost-utility analyses evaluated healthcare interventions for newborns (e.g. vaccination programs). Another recent review of HRQoL measurements (including generic and disease-specific instruments) in children and adolescents by Solans et al. confirmed that few studies measured utilities in paediatric populations [5]. Out of the 94 HRQoL instruments for children and adolescents reviewed in this publication, the HUI was cited once and no study used the EQ-5D questionnaire or the TTO or the SG method.

When performing a cost-utility analysis in a paediatric population, and in the absence of primary utility data (e.g. derived from a trial), the analyst is faced with a difficult question regarding the determination of the utilities. Although expert opinion has been commonly used in cost-utility analyses of paediatric interventions, judgment values have several limitations. On the other hand, direct measurements are time and resource intensive while most self-administered questionnaires are not applicable to a non-adult population. Furthermore, for young children who may not have the cognitive ability to answer questionnaires or participate in an interview, proxies need to be used. Another approach that has been used is to estimate utilities from adult patients. To gain a better understanding of the use of these methods in paediatric populations and to inform future cost-utility analyses in these populations, we systematically reviewed the published literature reporting utilities derived from direct (i.e. TTO and SG) and indirect (i.e. EQ-5D and HUI) measurements across conditions common in paediatric and adult populations.

## Methods

Studies presenting utility values derived from HUI or EQ-5D or using TTO or SG techniques in children and/or adult populations from randomized controlled trials (RCTs), comparative or non-comparative observational studies, or cross-sectional studies were included in the review. Although utilities can be derived from other questionnaires such as the SF-12 [6], the SF-6D [7] or the newly developed Assessment of Quality of Life (AQoL) [8], our search focussed on the HUI and EQ-5D and these two instruments are the most commonly used utility instruments for economic evaluations [4,9]. The search was limited to selected chronic diseases/conditions common to both children and adults [10,11], and known to have a decremental impact on health-related quality of life. These included skin diseases and asthma, two highly prevalent conditions in children and adults, as well as cancer and diabetes. Although less prevalent than asthma or skin diseases, cancer and diabetes seriously impact HRQoL and were included as well. Studies evaluating patients with chronic diseases were also included if the study population had patients with one of the above mentioned diseases. While the literature search strategy identified studies related to diabetes mellitus and all types of cancer, studies were excluded if they assessed only patients with type 2 diabetes or if the type of cancer affects only adults (e.g. colorectal, breast) since no comparison can be made with a paediatric population. Studies using the EQ-5D visual analogue scale (VAS) alone were excluded as the value derived from a VAS cannot directly be used as a utility without a transformation.

An electronic search of Ovid MEDLINE (1950-present), EMBASE (1980-present), and the Cochrane Library (via Wiley) was conducted to identify relevant citations published up to November 2008. A search strategy was developed for each electronic database using specific subject headings in addition to relevant text keywords. The detailed search strategies are shown in Additional file 1, Table S1: Electronic Database Search Strategies. No language restrictions were placed on the database searches. Study citations were downloaded into a Reference Manager 11^® ^database and all duplicate citations were identified and removed. One reviewer screened titles, abstracts and full-text versions of identified studies to determine study eligibility. A QUORUM diagram was used to summarize the study selection process. Data abstraction was completed by one reviewer and all data collected was verified by a second reviewer.

Included studies were classified based on the disease/condition, the population (children, adults, both), the type of utility measurement (EQ-5D, HUI, TTO, SG) and the level of evidence (RCT, observational, longitudinal, cross-sectional). The difference in utility gains/losses over time was captured for all prospective studies (e.g. utility at study end versus utility as study start). Where the change in utilities over time was not available (e.g. cross sectional studies), the mean utility values were recorded. Comparisons between children/adolescents and adults were assessed for studies evaluating both populations. Given the heterogeneity of included studies in terms of disease, population, and study design, the results of the literature review were summarized using a narrative approach. Similarly, the quality of individual studies was not assessed due to the heterogeneity of study designs, as there is no single tool available to evaluate the methodological quality of RCTs, non-randomized trials, cross-sectional studies and population health surveys. For the purposes of this study, subjects aged 18 years or less were defined as children/adolescents.

## Results

**Figure 1:**
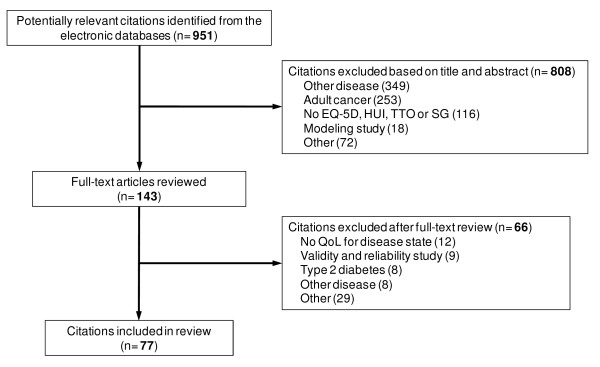
**Flow diagram for review of utilities derived using EQ-5D, HUI, TTO and SG**.

The literature search identified 951 citations of which 808 were excluded based on title and abstract screening. Out of the 143 studies which underwent full text review, 66 were excluded resulting in 77 studies included in our review. A flow diagram presenting information about the number of studies identified, included and excluded, and reasons for exclusion is shown in Figure 1. Table 1 presents an overview of studies included in our review in terms of medical condition, population, utility measure and study design.

**Table 1:** Summary of Included Studies (n = 77)

Condition	Population	No. of studies*	Utility Measure	Study Design (No. of studies*)
Asthma	Adults & children/adolescents	5	EQ-5D	RCT (1)
			HUI	cross-sectional (3)
			SG	cross-sectional (1)
	
	Children/adolescents	1	HUI	non-randomized cohort (1)
			SG	non-randomized cohort (1)
	
	Adults	19	EQ-5D	non-randomized cohort (2); cross-sectional (9)
			HUI	cross-sectional (4)
			SG	cohort (2); cross-sectional (3)
			TTO	cross-sectional (4)

Cancer	Adults & children/adolescents	11	HUI	cross-sectional (11)
	
	Children/adolescents	12	HUI	non-randomized cohort (4); cross-sectional (8)
			SG	cross-sectional (1)
			TTO	cross-sectional (1)

Chronic disease	Children/adolescents	2	HUI	cross-sectional (2)
			TTO	cross-sectional (1)

Diabetes	Children/adolescents	1	EQ-5D	non-randomized cohort (1)
	
	Adults	10	EQ-5D	non-randomized cohort (1); cross-sectional (5)
			HUI	cross-sectional (1)
			SG	cross-sectional (1)
			TTO	cross-sectional (3)

Skin diseases	Adults & children/adolescents	2	EQ-5D	cross-sectional (1)
			HUI	cross-sectional (1)
	
	Children/adolescents	2	SG	cross-sectional (1)
			TTO	cross-sectional (1)
	
	Adult	15	EQ-5D	RCT (4); non-randomized (1); cross-sectional (2)
			SG	cross-sectional (2); cohort (1); test-retest cohort (1)
			TTO	cross-sectional (5); cohort (1); pre-post (1)

EQ-5D-EuroQol-5Dimension; HUI-Health Utilities Index; SG-standard gamble; TTO-time trade off; RCT-randomized controlled trial. *Total number of studies is greater than 77, as some studies evaluated multiple conditions and/or used multiple methods of utility measurement.

**Figure 2:**
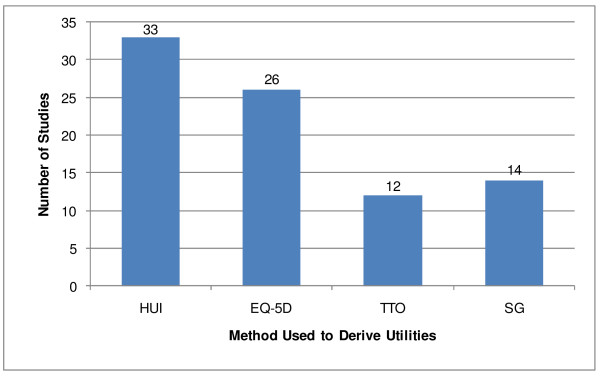
**Number of studies using direct and indirect measurements of utilities (n = 85)**. EQ-5D-EuroQol-5Dimension; HUI-Health Utilities Index; SG-standard gamble; TTO-time trade off; * Total number of studies is greater than 77, as some studies used multiple methods of utility measurement.

Overall, 21% of the studies evaluated both adults and children (n = 16), 23% evaluated children (n = 18) and 56% of the included studies evaluated an adult population only (n = 43). Direct measurements (i.e., SG, TTO) were used 31% of the time, while utilities were estimated using indirect methods (i.e., EQ-5D, HUI) 69% of the time (Figure 2). Although there were a higher proportion of studies using the HUI instrument compared to the EQ-5D instrument, the HUI instrument was primarily used in the evaluation of cancer patients. The results of selected studies are discussed by condition in the following sections. Each section begins by an overall overview of the identified studies, followed by a brief description of the studies starting with studies using indirect measurements (e.g. using the EQ-5D or the HUI) and then studies using direct measurements methods (e.g. TTO or SG). Further details of all included studies are shown in Additional files 2, 3, 4, 5, 6 (Tables S2-S6).

### Asthma

Of the 25 studies that reported utility values in patients with asthma, 5 included children and adults [10,12-15], one study evaluated children alone [16], and the remaining 19 studies evaluated adults only [17-35] (Additional file 2, Table S2-Utilities derived for asthma).

One study conducted in the Netherlands by Willems et al. [15], administered the EQ-5D instrument to children and adults enrolled into a RCT examining the effect of nurse-led telemonitoring versus usual outpatient care over 12 months. Results indicated that children and adults in the control groups had a similar improvement in EQ-5D utility of 0.01 points during the 12-month follow-up (from 0.78 (SD 0.17) to 0.79 (SD 0.21) for adults and from 0.96 (SD 0.07) to 0.97 (SD 0.05) for children). Although a change of 0.01 points is not considered a clinically important difference [36], this study suggests a similar gain in utilities between children and adults in an asthmatic population treated with usual care. The same study also showed that the gain in utility observed in the intervention group was higher in the paediatric population than in the adult population. However, it is unknown if these results reflect the fact that this nurse-led telemonitoring program was not effective in adults or if adults coped better with the disease than children.

The other four studies reporting utility data in children and adults with asthma had a cross-sectional study design and were undertaken in the US or Canada [10,12-14]. In the 1999 study by Mittmann et al., results indicated that HUI utility scores in asthmatic patients 12-19 years of age (mean: 0.90; SD 0.12) were similar to that of patients 20 to 29 years old (mean: 0.91; SD 0.11). Utility values associated with asthma decreased with the age of patients (e.g. 0.84 for 40-49 years of age and 0.76 for 60-69 years of age). Two other studies conducted in Canada reported utilities of 0.87 to 0.96 for asthmatic patients aged 12 years and over, using the HUI instrument. While the data was also collected through a national health survey, no breakdown by age or disease severity was provided. In the study by Chiou et al. [12], results were reported separately for cohorts of patients with a mean age of 9 years and a mean age of 38 years using the SG technique. SG utilities for a health state with moderate symptoms were higher for the adult cohort compared with children (0.96 versus 0.79), suggesting a greater impact of the disease on children.

Juniper et al. [16] evaluated utilities in a younger population (mean age: 12 years) that were recruited from a paediatric asthma clinic in Canada using the HUI instrument and SG technique. Results indicated differences between methods as the mean utility values were 0.89 (SD 0.09) using the HUI instrument and 0.82 (SD 0.15) using the SG technique. However, the mean values were close to the mean HUI and SG utility values of 0.90 and 0.79 reported by children and adolescents in the studies by Mittman et al. [10] and Chiou et al. [12], respectively.

Of the 19 studies reporting utility values associated with asthma in adults, 14 studies used an existing preference-based instrument (e.g. EQ-5D and/or HUI) [17,19,20,23-25,27-29,31-35] and utilities ranged from 0.33 to 0.92 reflecting different populations, disease severity or study settings. In general, studies found that adult patients with poor control of their disease had a lower quality of life [26,28,29,35].

### Cancer

Twenty-three studies estimated utility values associated with cancer using the HUI2 or HUI3 instrument (Additional file 3, Table S3-Utilities derived for cancer) [14,37-58]. Eleven studies evaluated children and adults using a cross-sectional study design. With the exception of one study which captured cancer utility data from a national health survey, the other 10 studies determined the utilities in survivors of childhood cancer at different survival time periods (e.g. 1 year, 10 years). Of the 12 studies which included children, 4 evaluated patients enrolled in non-randomized trials who were undergoing treatment for cancer [38,42,48,58], while 8 studies used a cross-sectional study design to evaluate children with cancer or children who had survived cancer [39,40,46,47,50,54,56,57].

Comparisons between the cancer studies are not straightforward given the vast differences in patient characteristics, evaluation periods, cancer types and treatment patterns. Despite differences in the type of cancer and the follow-up period, the majority of studies reported mean utility values greater than 0.8 for survivors of childhood cancer. Survivors of childhood acute lymphoblastic leukemia or Hodgkin's disease showed utility values ranging from 0.72 to 0.91 and from 0.75 to 0.88, respectively, whereas lower utility values were reported for survivors of germ cell tumours (mean: 0.49) and retinoblastomas (mean: 0.51-0.78). Five studies evaluating utilities using different proxies such as parents, physicians or nurses [38,43,45,46,52] showed marked differences in results obtained from different assessors.

### Chronic disease

Two studies were identified that determined utility values of children and adolescents with chronic conditions [59,60]. The aim of these two studies was to examine the difference in utility estimates dependent on whether the children themselves or their parents/paediatricians were the assessors. A comparison of the HUI2, HUI3, and TTO scores by Sung et al. [60] indicated that for both parents and children, the utilities were higher with the HUI2 (which was specifically developed for use in children) while the utilities derived from the HUI3 or TTO experiments were similar. In addition, this study showed that utilities derived from children were higher than those derived from their parents. In another study [59], utilities derived from paediatricians (mean: 0.93) were higher than those derived from parents (mean: 0.80). In both studies, utilities derived from parents were similar in magnitude. Details are presented in Additional file 4, Table S4-Utilities derived for chronic disease.

### Type 1 diabetes mellitus

Although eleven studies reported utility data associated with type 1 diabetes mellitus [61-71], only one study included children (Additional file 5, Table S5-Utilities derived for diabetes) [70]. In a postal survey of children enrolled in a prospective cohort study, Nordfeldt et al. [70] demonstrated that patients with severe hypoglycemia had a median utility of 0.85 and patients without severe hypoglycemia had a median EQ-5D utility of 1.0, however, no further details were given in this study regarding this result (i.e. median utility of 1.0).

Ten studies collected utility data among adults with type 1 diabetes mellitus. One study employed a cohort design [66], while the remaining studies had a cross-sectional study design [61-65,67-69,71]. Among all the studies in adults, the reported utility with preference-based instruments (i.e. EQ-5D) ranged from 0.52 to 0.90 reflecting difference in study settings and patients' disease severity. Four studies [61,62,67,68] using a cross-sectional study design used either the TTO method or the SG method in adults with type I diabetes mellitus. These studies were carried out in the US, UK and Canada. In the studies by Brown et al. [61], Chancellor et al. [62], and Landy et al. [68], the results of the TTO approach were of the same order of magnitude across studies with a mean utility value of 0.88 (SD 0.117), 0.83 (SD 0.02), and 0.873, respectively.

### Skin diseases

Utility data were reported in 19 studies [10,34,72-88] conducted in the area of skin diseases, of which 15 evaluated an adult population (Additional file 6, Table S6-Utilities derived for skin disease). Two studies evaluated the utility values for both children/adolescents and adults with acne [10,76], The EQ-5D was administered to 54 dermatology clinic patients with severe acne who were at least 16 years of age (mean age: 22 years) [76]. In this prospective, non-randomized study, patients' mean utilities increased from a value of 0.84 (standard deviation (SD) 0.17) at baseline to 0.93 (SD 0.15) after 12 months of acne treatment. However, data was not presented separately for children and adults. In the 1999 study by Mittmann et al., utility values were presented for specific conditions (e.g. acne, asthma) using data from 17,626 Canadians aged 12 to 80+ years who participated in the Canadian Community Health Survey (CCHS) conducted by Statistics Canada [10]. Among other questions, the CCHS included the HUI instrument. Results indicated that HUI utility scores in acne patients 12-19 years of age (mean: 0.92; SD 0.90) were similar to that of patients 20 to 29 years old (mean: 0.92; SD 0.09).

One study determined the utility of children/adolescents with skin disease using the SG technique by deriving preferences from the general public (mean age: 54 years). In this study, the utility value associated with children with atopic dermatitis was estimated at 0.84 [85]. In another cross-sectional study of 266 adolescents with acne conducted at four US high schools, utilities were estimated to be 0.96 (SD 0.092), based on a TTO approach [73].

Fifteen studies reported utility data collected in adults. Of the 7 studies that assessed the quality of life of adults with skin disease by applying a preference-based instrument [34,72,79,83,84,86,87], all 7 studies used the EQ-5D questionnaire and 6 of these studies examined HRQoL of patients with psoriasis. In these 6 studies, the mean utility ranged from 0.66 to 0.80. Two RCTs that provided utility data at baseline and after several weeks of follow-up, during which patients were treated with either placebo or an active agent, indicated an increase of 0.2 utility points following 12 weeks of treatment, which included both responders and non-responders to treatment [79,84].

Eight studies used direct estimation techniques to evaluate adult patients' utilities associated with skin diseases [74,75,77,78,80-82,88], with the majority of these studies (n = 5) assessing patients with psoriasis. These studies evaluated adult patients with a mean age between 28 and 54 years. With the exception of the 3 studies by Littenberg et al. [77], Lundberg et al. [78] and Schiffner et al. [81], the other 5 studies were cross-sectional studies. Although not a prospective study, one study evaluated the utility associated with treatment response. Based on 58 patients undergoing treatment at a dermatology outpatient clinic, Schmitt and colleagues found a difference of 0.43 utility points between patients in whom psoriasis was controlled by their treatment versus non-responders, while a difference of 0.31 utility points between responders and non-responders was shown in eczema patients [82]. The impact of disease severity was assessed in a sample of psoriasis patients from a tertiary medical centre using both TTO and SG methods in the study by Zug et al. [88], which demonstrated a decrease in mean utility values with higher proportions of body surface area affected by psoriasis.

## Discussion

In this review, we identified 77 studies which reported utility values across conditions that are common in paediatric and adult populations. Although the majority of these studies evaluated utilities in adult populations, 23% of the studies evaluated utilities in children and a similar proportion (i.e. 21%) evaluated utilities for both children/adolescents and adults. When measuring utilities, pre-existing instruments (e.g. HUI, EQ-5D) were used in two-thirds of the studies. Few studies provided utilities over time or by response type (e.g. responder to treatment versus non-responder), which are often required in economic modelling.

The majority of the studies conducted in children were among cancer patients and there is a paucity of utility data for children living with other conditions. As such, in the absence of primary data, proxies may be used. Although few studies provided comparative information on utility values between children and adults, a few trends emerged. The study conducted by Mittmann et al. suggested that utility values between adolescents (e.g. 12-20 years of age) and young adults (e.g. 20-29 years of age) suffering from acne or asthma was similar [10]. While limited by small sample sizes, other studies in the area of acne also suggested similar utility values between children and adults. Results of the only RCT reporting utility values over time in children and adults indicated similar utility gains between children and adults with asthma who received usual care while a higher utility gain was observed among children in the intervention group [15].

The results also suggested that different methods may lead to different utility values. While some studies, such as Sung et al. [60], demonstrated somewhat similar utilities derived from the HUI-2, HUI-3, and TTO methods in children and adult patients with chronic disease (range 0.92-0.95), the study by Moy et al. [29] showed differences in utility when using HUI-3, SG or TTO techniques (0.57, 0.91, 0.81, respectively) in a cohort of patients with asthma. It has been shown that different methods used to collect utility data may yield different HRQoL values in the same group of patients [89]. The results of the three studies that used SG and TTO methods in the same group of patients [18,29,54] supported our assumption that the SG method tends to yield higher utility values than the time trade-off method [89].

Different types of assessors (e.g. parents versus children [60] or parents versus paediatricians [59]) used in the estimation of utilities also led to differences in utilities. Studies comparing utilities between patients and proxies were common in the area of cancer. Those studies typically used parents, physicians and nurses as a proxy. It has been argued that the use of medical staff and teachers as proxies may give biased estimates when determining utility values, since their rating of some dimensions of HRQoL differ from the one of children assessing their own health [90]. The rating of parents for example may be affected by their knowledge about health and health care and by their own current health status [91].

Limitations associated with this review can be linked to the broad research question (i.e. utilities in children and adults), and the challenges associated with developing a literature search strategy that included all relevant studies. Thus, there is a risk that some studies may have been missed in the initial screening process as only one reviewer screened the data. To minimize this risk, all the references listed in the included studies, reviews, commentaries or letters were manually searched to identify potential studies. The grey literature was not searched and unpublished utility evaluations may be available through online services but were not included in our review. Another limitation of our review is that we did not assess the quality of the different studies, but instead reviewed the main results and conclusions as stated by the authors to determine some common trends or directions among the various studies. It was beyond the scope of this review to critically appraise the methodological quality of studies. In the comparison of utility values between children and adults, some differences in results may be due to chance or due to the methods not being used correctly or consistently. The relatively small sample size of some of the studies may compromise the validity of the results. Small sample sizes in HRQoL studies have also been reported elsewhere [92]. Comparisons of utilities between children and adults were especially difficult to assess in those studies evaluating patients with cancer and diabetes due to important differences in patient characteristics (e.g. cancer types), study design (e.g. evaluation period) or interventions. The majority of these studies were cross-sectional, limiting our understanding of gain in utility values over time which is almost always required for economic evaluations. We restricted our search to specific utility instruments and conditions. For example, we did not include the newly developed AQoL or SF-6D. Finally, our review was limited to asthma, skin diseases, type I diabetes, certain types of cancer common to both children and adults, and overall chronic conditions, which may not represent the whole body of literature that reports utility data in children or adolescents. Expanding the search to other conditions or diseases common to both children and adults that have a negative impact on HRQoL (e.g. epilepsy) is left for future research. However, it is unlikely that the trends observed in our studies would change by the expansion of this review to other conditions common to children and adults.

Despite these limitations, this review identified 77 studies reporting utility values derived from direct (SG or TTO techniques) or indirect (pre-existing questionnaires such as the EQ-5D and HUI) measurements across conditions common to children and adults, that could be used for future reference or for the conduct of sensitivity analyses in economic evaluations. The findings of this review showed that the previous research on utilities of children has primarily focused in the collection of utilities in cancer patients (12 out of 18 studies) which may be related to the development and validation of the HUI-2. This review also indicated that few studies have been conducted to estimate the utilities related to children with asthma, diabetes or skin diseases. Although there are no studies to compare our findings with, our review complements the recent review of generic and disease specific instruments in children and adolescents [5] by identifying studies reporting utilities in children and adults with asthma, cancer, chronic disease, type 1 diabetes and skin diseases.

## Conclusions

When interventions have an impact on HRQoL, utility data are increasingly being used in economic evaluations of health care technologies as they are required to calculate QALYs in these studies. As such, reliable utility data is therefore needed. As shown in this review of 77 studies, few studies have been set up to collect utilities in children and adolescents, with the exception of studies evaluating utilities in cancer patients. Canadian health surveys have shown that utilities between adolescents and young adults were similar in magnitude, suggesting that in lack of better data, utility data obtained from young adult populations may be used as a proxy for utilities in children. Nevertheless, other studies have shown that utility values differed when using different estimation methods.

In light of these results, researchers in paediatric medicine should be encouraged to conduct utility measurements in their patients. This would increase the availability of utility data in paediatric patients and possibly provide a greater understanding of the methodological issues that are still present. For the time being, analysts who conduct economic evaluations of interventions among children or adolescents should conduct comprehensive sensitivity analyses regarding the impact of the utility values on their cost-effectiveness estimates.

## Competing interests

The authors declare that they have no competing interests.

## Authors' contributions

JET conceived the study and its design, analyzed and interpreted the data and wrote the manuscript. NB participated in the data acquisition, data analysis, and the writing and editing of the manuscript. MB was involved in the design of the study, interpretation of the data, and drafting the manuscript. RBH participated in the data collection, analysis and interpretation of data. LG was involved in the data collection and preparation of the data tables. KC contributed to the design of the study and participated in the data acquisition. FX, DOR, RG contributed to the study design and critically revised the manuscript for important intellectual content. All authors have read and approved the final manuscript.

## Supplementary Material

Additional file 1 **Table S1: Electronic Database Search Strategies**. Table showing the electronic database search strategies, in PDF format. Click here for file

Additional file 2 **Table S2 - Utilities derived for asthma**. Table showing utilities derived for asthma, in PDF format. Click here for file

Additional file 3 **Table S3 - Utilities derived for cancer**. Table showing utilities derived for cancer, in PDF format. Click here for file

Additional file 4 **Table S4 - Utilities derived for chronic disease**. Table showing utilities derived for chronic disease, in PDF format. Click here for file

Additional file 5 **Table S5 - Utilities derived for type 1 diabetes mellitus**. Table showing utilities derived for type 1 diabetes mellitus, in PDF format. Click here for file

Additional file 6 **Table S6 - Utilities derived for skin disease**. Table showing utilities derived for skin disease, in PDF format. Click here for file
