# Supplementary material for: A review of health utilities across conditions common in paediatric and adult populations
Source: Health Qual Life Outcomes. 2010 Jan 27;8:12. doi: 10.1186/1477-7525-8-12 (PMC2828427; doi:10.1186/1477-7525-8-12)
Supplement: Additional file 1 — Table S1: Electronic Database Search Strategies. Table showing the electronic database search strategies, in PDF format. [file 1477-7525-8-12-S1.PDF]

Additional File 1: Electronic Database Search Strategies

Ovid MEDLINE and EMBASE:

1 (euroqol$ or euro qol$ or eq5d or eq 5d).mp.
2 (hui or hui1 or hui2 or hui3 or huir or health utilities index$ or health utilities indic$ or health utility index$ or health utility indic$).mp.
3 time trade off/
4 (time trade off? or time tradeoff? or TTO?).ti,ab.
5 standard gamble$.ti,ab.
6 1 or 2 or 3 or 4 or 5
7 exp Skin Disease/ use emez
8 exp Skin Diseases/ use mesz
9 (psoriasis or skin disease$).ti.
10 exp Asthma/
11 asthma$.ti.
12 exp Acneiform Eruptions/
13 acne.ti.
14 exp Acne/
15 exp Neoplasms/ use emez
16 exp Neoplasm/ use mesz
17 (cancer$ or neoplasm$ or tumor$).ti.
18 exp Diabetes Mellitus/
19 (diabetes or diabetic).ti.
20 7 or 8 or 9 or 10 or 11 or 12 or 13 or 14 or 15 or 16 or 17 or 18 or 19
21 6 and 20

Cochrane Library (via Wiley):

#1 (euroqol* OR "euro qol*" OR eq5d OR "eq 5d"):ti,ab,kw
#2 (hui OR hui1 OR hui2 OR hui3 OR huir OR "health utilities index*" OR "health utilities indic*" OR "health utility index*" OR "health utility indic*"):ti,ab,kw
#3 (time trade off* or time tradeoff* or TTO*):ti,ab
#4 standard gamble:ti,ab
#5 (#1 OR #2 OR #3 or #4)
#6 MeSH descriptor **Skin Diseases** explode all trees
#7 (psoriasis OR skin disease*):ti
#8 MeSH descriptor **Asthma** explode all trees
#9 (asthma*):ti
#10 MeSH descriptor **Acneiform Eruptions** explode all trees
#11 (acne):ti
#12 MeSH descriptor **Diabetes Mellitus** explode all trees
#13 (diabetes OR diabetic):ti
#14 MeSH descriptor **Chronic Disease** explode all trees
#15 MeSH descriptor **Neoplasms** explode all trees
#16 (cancer* OR neoplasm* OR tumor*):ti
#17 (#6 OR #7 OR #8 OR #9 OR #10 OR #11 OR #12 OR #13 OR #14 OR #15 OR #16)
#18 (#5 AND #17)
