# Supplementary material for: A review of health utilities across conditions common in paediatric and adult populations
Source: Health Qual Life Outcomes. 2010 Jan 27;8:12. doi: 10.1186/1477-7525-8-12 (PMC2828427; doi:10.1186/1477-7525-8-12)
Supplement: Additional file 2 — Table S2 - Utilities derived for asthma. Table showing utilities derived for asthma, in PDF format. [file 1477-7525-8-12-S2.PDF]

Table S2 - Utilities derived for asthma

Author, Year, Country	Study Design	Interventions	Setting	Mean (SD) Age	% Males	Utility Instrument	Baseline Utility		End of Study Utility	
							N	Mean (SD)	N	Mean (SD)
**Children/Adolescents & Adults**										
Willems et al.<br>2007<br>Netherlands	RCT;<br>12 months	nurse-led telemonitoring program vs usual outpatient care	Outpatient clinic	Adults control: 45.9 (15.9)<br>Adults intervention: 45.7 (11.3)<br>Children control: 10.9 (2.3)<br>Children intervention: 10.6 (2.1)	33.3<br>42.3<br>55.6<br>72.4	**EQ-5D index**	Adults control: 27<br>Adults intervention: 26<br>Children control: 27<br>Children intervention: 29	0.78 (0.17)<br>0.89 (0.13)<br>0.96 (0.07)<br>0.92 (0.20)	Adults control: 27<br>Adult intervention: 26<br>Children control: 27<br>Children intervention: 29	0.79 (0.21)<br>0.90 (0.11)<br>0.97 (0.05)<br>0.98 (0.04)
Mittmann et al.<br>1999<br>Canada	Cross-sectional	n/a	National health survey (n=17,626)	n/a (age ≥12)	45.7	**HUI 3**	Overall: 1128<br>Age 12-19: 220<br>Age 20-29: 221<br>Age 30-39: 207<br>Age 40-49: 159<br>Age 50-59: 104<br>Age 60-69: 105<br>Age 70-79: 77<br>Age >80: 29<br>No comorbidities: 229	0.86 (0.17)<br>0.90 (0.12)<br>0.91 (0.11)<br>0.88 (0.15)<br>0.84 (0.18)<br>0.80 (0.19)<br>0.76 (0.21)<br>0.78 (0.19)<br>0.74 (0.26)<br>0.92 (0.097)	n/a	n/a
Mittmann et al.<br>2001<br>Canada	Cross-sectional	n/a	National health survey (n=47,534)	Age 12-19 : 12.3%<br>Age >19: 87.7%	51.8	**HUI 3**	Asthma: 897	0.955 (0.066)	n/a	n/a
Schultz & Kopec<br>2003<br>Canada	Cross-sectional	n/a	National health survey (n=73,402)	n/a (age ≥12)	49.2	**HUI 3**	Asthma: 5467	0.87	n/a	n/a
Chiou et al.<br>2005<br>USA	Cross-sectional	n/a	Community survey of 114 adults;<br>72 children enrolled in RCT	Adults: 38 (15.4)<br>Children: 9.3 (range 7-12)	42<br>n/a	**SG**	Adults: 114<br>moderate symptoms<br>emotional symptoms<br>activity limitations<br>Children: 72<br>mild symptoms<br>moderate symptoms<br>severe symptoms	 0.96<br>0.89<br>0.85<br><br>0.79<br>0.70<br>0.28	n/a	n/a
**Children/Adolescents**										
Juniper et al.<br>1997<br>Canada	Non-randomized, prospective cohort;<br>9 weeks	none	Pediatric asthma clinic	12.0 (3.1)	57.7	**HUI**	52	0.89 (0.09)	n/a	n/a
						**SG**	40	0.82 (0.15)	n/a	n/a
**Adults**										
Aburuz et al.<br>2007<br>UK	Cross-sectional	n/a	Asthma specialist clinic	42.3 (15.0)	38.4	**EQ-5D index**	86	0.47 (0.33)	n/a	n/a
Burstrom et al.<br>2001<br>Sweden	Cross-sectional	n/a	National health survey (n=3,112)	Men: 48.7;<br>Women: 49.0	45.3	**EQ-5D index**	Asthma: 253	Overall: 0.79 (0.015)<br>Male: 0.80 (0.027)<br>Female: 0.78 (0.017)	n/a	n/a
Chen et al.<br>2007<br>USA	Cross-sectional data from prospective cohort study	none	National cohort	52.8	27	**EQ-5D index**	not done	not done	987	0.86 (0.16)
Ko & Coons<br>2006<br>USA	Cross-sectional	n/a	General population survey (n=4,048)	44.7 (17.4)	48	**EQ-5D index**	Asthma: 68	0.924 (0.0117)	n/a	n/a
Lloyd et al.<br>2007<br>UK	Non-randomized, prospective cohort;<br>4 weeks	none	Outpatient clinics & primary care offices	No exacerbation: 40.5 (11.6)<br>Exacerbation: 41.4 (12.0)<br>Exacerbation with hospitalization: 48.4 (11.0)	39.3<br>27.3<br>40	**EQ-5D index**	n/a	n/a	No exacerbation: 85<br>Exacerbation: 22<br>Exacerbation with hospitalization: 5	0.89 (0.15)<br>0.57 (0.36)<br>0.33 (0.39)
Lubetkin et al.<br>2005<br>USA	Cross-sectional	n/a	General population survey (n=13,646)	n/a	43	**EQ-5D index**	1202	0.82 (0.0069)	n/a	n/a

Oga et al.<br>2003<br>Japan	Non-randomized, prospective,<br>patients undergoing therapeutic regimen; 6 months	n/a	Outpatient	46.8 (19.3)	41	**EQ-5D index**	54	0.808 (0.187)	54	0.879 (0.146)
Polley et al.<br>2008<br>Ireland	Cross-sectional	n/a	Respiratory outpatient clinic	51.6 (17.5)	65	**EQ-5D index**	20	0.63 (0.38)	n/a	n/a
Saarni et al.<br>2006<br>Finland	Cross-sectional	n/a	General population survey (n=6,681)	52.6	47	**EQ-5D index**	Asthma: 8.8% of population	0.766 (0.011)	n/a	n/a
Leidy et al.<br>1998<br>USA	Cross-sectional	n/a	Population-based survey of low-income adults with asthma	Sample: 33.4 (9.4)<br>African American: 33.1 (8.0)<br>Caucasian: 33.6 (10.4)	25.9<br>37.0<br>18.2	**HUI**	Total Sample: 112<br>African American: 46<br>Caucasian: 66	0.80 (0.16)<br>0.78 (0.17)<br>0.80 (0.16)	n/a	n/a
Revicki et al.<br>1998<br>USA	Cross-sectional	n/a	Hospital clinic patients	34.7 (10.7)	41	**HUI 2**	161	0.84 (0.17)	n/a	n/a
Flood et al.<br>2006<br>France, Italy, UK, USA	Cross-sectional	n/a	Community	45	44	**SG**	USA: 161<br>France: 30<br>Italy: 30<br>UK: 30	USA: 0.86 (0.14)<br>France: 0.86 (0.17)<br>Italy: 0.86 (0.17)<br>UK: 0.76 (0.19)	n/a	n/a
Juniper et al.<br>2001<br>Canada	Cohort, prospective	none	Community	38 (11)	30	**SG**	40	0.87 (0.13)	n/a	n/a
Lloyd et al.<br>2008<br>UK	Cohort, prospective	none	Community	40.0 (12.5)	37.8	**SG**	Sample: 88<br>Complete control<br>Marked improvement<br>Limited improvement<br>No change<br>Worsening	 0.784 (0.06)<br>0.756 (0.06)<br>0.748 (0.061)<br>0.705 (0.064)<br>0.711 (0.063)	n/a	n/a
Nyman et al.<br>2007<br>USA	Cross-sectional	n/a	National health survey (n=3504)	n/a (age ≥18)	n/a	**TTO**	Asthma: 3504	Overall: 0.820<br>Age 18-24: 0.895<br>Age 25-34: 0.879<br>Age 35-44: 0.832<br>Age 45-54: 0.794<br>Age 55-64: 0.768<br>Age 65-74: 0.756<br>Age 75+: 0.696	n/a	n/a
Blumenschein & Johannesson<br>1998<br>USA	Cross-sectional	n/a	Community pharmacy, hospital and outpatient clinic	40.3 (15.1)	25	**SG**	69	0.91 (0.018)	n/a	n/a
						**TTO**	69	0.89 (0.019)	n/a	n/a
McTaggart-Cowan et al.<br>2008<br>Canada	Cross-sectional	n/a	Self-reported asthma patients recruited from poster advertisement	35.0 (7.9)	30	**EQ-5D index**	Overall: 157<br>Asthma severity:<br>very mild: 21<br>mild: 59<br>moderate: 51<br>severe: 20<br>Asthma control:<br>very well controlled: 37<br>well controlled: 43<br>adequately controlled: 54<br>not well controlled: 19	0.84 (0.23)<br><br>0.84 (0.29)<br>0.89 (0.18)<br>0.81 (0.21)<br>0.76 (0.27)<br><br>0.90 (0.22)<br>0.84 (0.20)<br>0.81 (0.22)<br>0.80 (0.21)	n/a	n/a

						**HUI 3**	Overall: 157<br>Asthma severity:<br>very mild: 21<br>mild: 59<br>moderate: 51<br>severe: 20<br>Asthma control:<br>very well controlled: 37<br>well controlled: 43<br>adequately controlled: 54<br>not well controlled: 19	0.84 (0.20)<br><br>0.82 (0.22)<br>0.88 (0.18)<br>0.84 (0.15)<br>0.75 (0.27)<br><br>0.88 (0.18)<br>0.83 (0.20)<br>0.84 (0.15)<br>0.84 (0.16)	n/a	n/a
Moy et al.<br>2004<br>USA	Cross-sectional	n/a	Pharmacy database	49 (15)	24	**HUI 3**	Overall: 100<br>Mild: 41<br>Moderate: 33<br>Severe: 26	0.57 (0.34)<br>0.61 (0.35)<br>0.58 (0.35)<br>0.49 (0.34)	n/a	n/a
						**SG**	Overall: 100<br>Mild: 41<br>Moderate: 33<br>Severe: 26	0.91 (0.15)<br>0.92 (0.15)<br>0.93 (0.14)<br>0.86 (0.17)	n/a	n/a
						**TTO**	Overall: 100<br>Mild: 41<br>Moderate: 33<br>Severe: 26	0.81 (0.22)<br>0.90 (0.15)<br>0.82 (0.24)<br>0.66 (0.22)	n/a	n/a
Szende et al.<br>2004<br>Hungary	Cross-sectional	n/a	Outpatients & inpatients	49	34	**EQ-5D index**	Good control: 36<br>Mildly reduced control: 64<br>Mod. reduced control: 82<br>Poor control: 46	0.93<br>0.76<br>0.65<br>0.52	n/a	n/a
						**TTO**	Good control: 36<br>Mildly reduced control: 64<br>Mod. reduced control: 82<br>Poor control: 46	0.99<br>0.96<br>0.82<br>0.73	n/a	n/a

SD-standard deviation; n/a-not available; RCT-randomized controlled trial
