# Supplementary material for: A review of health utilities across conditions common in paediatric and adult populations
Source: Health Qual Life Outcomes. 2010 Jan 27;8:12. doi: 10.1186/1477-7525-8-12 (PMC2828427; doi:10.1186/1477-7525-8-12)
Supplement: Additional file 3 — Table S3 - Utilities derived for cancer. Table showing utilities derived for cancer, in PDF format. [file 1477-7525-8-12-S3.PDF]

Table S3 - Utilities derived for cancer

Author, Year, Country	Study Design	Condition	Interventions	Setting	Mean (SD) Age	% Males	Utility Instrument	Baseline Utility		End of Study Utility	
								N	Mean (SD)	N	Mean (SD)
**Children/Adolescents & Adults**											
Alessi et al.<br>2007<br>Italy	cross-sectional	survivors of childhood cancer	n/a	postal questionnaire to cancer registry patients (n=644)	n/a; age >15	Range 30-73.7	**HUI 3**	Leukemia: 187<br>Non-Hodgkin lymphoma: 46<br>Hodgkin disease: 49<br>CNS tumours: 133<br>Neuroblastoma: 35<br>Retinoblastoma: 19<br>Wilms tumour: 42<br>Bone tumour: 31<br>Soft tissue sarcomas: 42<br>Gonadal tumours: 20<br>All other: 40	0.85<br>0.88<br>0.85<br>0.73<br>0.75<br>0.51<br>0.85<br>0.75<br>0.85<br>0.83<br>0.88	n/a	n/a
Cardarelli et al.<br>2006<br>Italy	cross-sectional	survivors of childhood cancer	n/a	previously treated patients (n=213)	13.3 (range 8-28)	52	**HUI 2**	Self assessment<br>Brain tumours: 50<br>Leukemia/lymphoma: 89<br>Other solid tumours: 74<br>Overall population: 213	 0.87<br>0.96<br>0.94<br>0.92	n/a	n/a
Felder-Puig et al.<br>2000<br>Germany	cross-sectional	survivors of childhood cancer	n/a	oncology outpatient department (n=142)	13 (range 6-30)	57.7	**HUI 2**	No sequelae<br>Patients/parents: 74<br>Physicians: 76<br>Nurses: 88<br>Mild sequelae<br>Patients/parents: 60<br>Physicians: 47<br>Nurses: 46<br>Moderate to severe sequelae<br>Patients/parents: 8<br>Physicians: 19<br>Nurses: 8	 0.94 (0.08)<br>0.97 (0.05)<br>0.96 (0.05)<br><br>0.88 (0.14)<br>0.95 (0.10)<br>0.93 (0.05)<br><br>0.80 (0.18)<br>0.78 (0.23)<br>0.64 (0.28)	n/a	n/a
Fluchel et al.<br>2008<br>Uruguay	cross-sectional	survivors of childhood cancer & age-matched controls	n/a	cancer registry	survivors: 13.6 (4.26)<br>controls: 12.2 (2.7)	49.5<br>33.3	**HUI 3**	Brain tumour: 19<br>ALL survivors: 49<br>All survivors: 92<br>Controls: 96	0.601 (0.2399)<br>0.722 (0.2496)<br>0.661 (0.2625)<br>0.80 (0.1429)	n/a	n/a
Fu et al.<br>2006<br>6 countries in Central America	cross-sectional	survivors of childhood cancer	n/a	previously treated patients (n=211)	median: 12.8<br>(range 3-25)	56	**HUI 2**	All patients self-assessment: 165<br>All patients parent proxy: 140<br>All patients physician proxy: 201<br>ALL: 72<br>Hodgkin's disease: 28	0.872 (0.16)<br>0.90 (0.12)<br>0.94 (0.11)<br>0.871 (0.18)<br>0.858 (0.17)	n/a	n/a
							**HUI 3**	All patients self-assessment: 175<br>All patients parent proxy: 181<br>All patients physician proxy: 200<br>ALL: 76<br>Hodgkin's disease: 30	0.733 (0.27)<br>0.84 (0.2)<br>0.89 (0.19)<br>0.725 (0.30)<br>0.747 (0.24)	n/a	n/a
Kennedy et al.<br>1999<br>UK	cross-sectional	survivors of brain tumours	n/a	previously treated patients	median: 8.6<br>(range 2-19)	n/a	**HUI 2**	32	0.81 (0.178)	n/a	n/a
Pogany et al.<br>2006<br>Canada	cross-sectional	survivors of primary cancer before age 20 & general population controls	n/a	patients identified through national retrospective cohort study	n/a (range 5-37)	survivors: 51.2<br>controls: 48.4	**HUI 3**	survivors: 2152<br>controls: 2432	0.83 (0.21)<br>0.87 (0.17)	n/a	n/a
Schultz & Kopec<br>2003<br>Canada	cross-sectional	cancer	n/a	National health survey (n=73,402)	n/a (age ≥12)	49.2	**HUI 3**	Cancer: 1359	0.78	n/a	n/a

Shimoda et al.<br>2008<br>Brazil	cross-sectional	survivors of childhood cancer	n/a	previously treated patients	22.8 (range 13-40)	54	**HUI 3**	All patients: 138<br>ALL: 31<br>Hodgkin disease: 15<br>Non-hodgkin lymphoma: 13<br>Osteosarcoma: 15<br>Retinoblastoma: 18<br>Wilms tumour: 23<br>Germ cell tumour: 6<br>Other: 17	0.80 (0.263)<br>0.83 (0.225)<br>0.76 (0.215)<br>0.88 (0.164)<br>0.80 (0.259)<br>0.78 (0.324)<br>0.78 (0.283)<br>0.49 (0.462)<br>0.89 (0.189)	n/a	n/a
Shimoda et al.<br>2005<br>Brazil	cross-sectional	survivors of childhood cancer	n/a	previously treated patients	23.4 (5.51)	60	**HUI 2**	Self-Assessment<br>All patients: 50<br>ALL: 8<br>Physician Proxy<br>All patients: 50<br>Nurse Proxy<br>All patients: 50<br>ALL patients: 8<br>Hodgkin's disease: 8<br>Wilms' tumour: 8	 0.84 (0.178)<br>0.88 (0.19)<br><br>0.84 (0.166)<br><br>0.80 (0.158)<br>0.85 (0.16)<br>0.88 (0.08)<br>0.81 (0.12)		
							**HUI 3**	Self-Assessment<br>All patients: 50<br>ALL: 8<br>Hodgkin's disease: 8<br>Physician Proxy<br>All patients: 50<br>Nurse Proxy<br>All patients: 50<br>ALL patients: 8<br>Hodgkin's disease: 8<br>Wilms' tumour: 8	 0.78 (0.271)<br>0.85 (0.25)<br>0.80 (0.21)<br><br>0.74 (0.307)<br><br>0.73 (0.280)<br>0.85 (0.17)<br>0.80 (0.21)<br>0.76 (0.24)	n/a	n/a
Van Schaik et al.<br>1999<br>Canada	cross-sectional	survivors of childhood Hodgkin's disease	n/a	previously treated patients	median: 21.9	66.7	**HUI 2**	33	0.85 (0.13)	n/a	n/a
**Children/Adolescents**											
Barr et al.<br>1997<br>Canada	Non-randomized, prospective;<br>3 weeks	ALL	chemotherapy on ambulatory basis	hospital	median: 3 yrs 11 months (range 11 months - 14 years)	n/a	**HUI 2**	Parents proxy: 18<br>Physician proxy: 18<br>Nurse proxy: 18	0.86 (0.17)<br>0.90 (0.10)<br>0.96 (0.05)	18<br>18<br>18	0.89 (0.09)<br>0.89 (0.12)<br>0.91 (0.13)
Barr et al.<br>1999<br>Canada	cross-sectional	survivors of CNS tumours	n/a	neuro-oncology follow up clinic	9.5 (range 1-17)	50	**HUI 2**	Disease free: 28<br>Residual: 10<br>Recurrent: 3	0.89 (0.13)<br>0.81 (0.19)<br>0.56 (0.41)	n/a	n/a
							**HUI 3**	Disease free: 28<br>Residual: 10<br>Recurrent: 3	0.78 (0.26)<br>0.56 (0.26)<br>0.32 (0.57)	n/a	n/a
Barr et al.<br>2000<br>Canada	cross-sectional	survivors of Wilm's tumour or neuroblastoma	n/a	previously treated patients	Wilm's: 9.1 (3.35)<br>Neuroblastoma: 7.8 (3.13)	50<br>31	**HUI 2**	Wilm's: 52<br>Neuroblastoma: 26	0.95 (0.08)<br>0.90 (0.13)	n/a	n/a
							**HUI 3**	Wilm's: 52<br>Neuroblastoma: 26	0.93 (0.12)<br>0.87 (0.19)	n/a	n/a
Cox et al.<br>2005<br>USA	Non-randomized, prospective; 31 weeks	ALL	institutional treatment protocol	hospital	median: 10<br>(range 6-18)	44.4	**HUI 3**	n/a	n/a	22	0.87 (SE=0.06)
Glaser et al.<br>1999<br>UK	cross-sectional	survivors of CNS tumours	n/a	previously treated patients	10.5 (range 6-16)	33.3	**HUI 2**	Survivors: 25<br>Parents: 26<br>Physicians: 23<br>Physiotherapist: 28	0.78 (0.18)<br>0.82 (0.18)<br>0.89 (0.11)<br>0.85 (0.13)	n/a	n/a
							**HUI 3**	Survivors: 26<br>Parents: 27<br>Physicians: 24<br>Physiotherapist: 28	0.66 (0.28)<br>0.72 (0.29)<br>0.83 (0.17)<br>0.76 (0.24)	n/a	n/a
Grant et al.<br>2006<br>Canada	cross-sectional	survivors of childhood cancer	n/a	previously treated patients	males: 17.55 (1.35)<br>females: 17.81 (1.51)	50	**HUI 2**	All patients: 84<br>Males: 42<br>Females: 42	0.87 (0.14)<br>0.90<br>0.83	n/a	n/a

							HUI 3	All patients: 84<br>Males: 42<br>Females: 42	0.79 (0.23)<br>0.73<br>0.84	n/a	n/a
Hinds et al.<br>2007<br>USA	Non-randomized, prospective; 48 weeks	ALL	treatment protocol for ALL	pediatric oncology units	median: 8.6<br>(range 4-18)	66	HUI 3	n/a	n/a	24	0.91 (0.03)
Merchant et al.<br>2002<br>USA	cross-sectional	survivors of craniopharyngioma	n/a	previously treated patients	median: 8.6<br>(range 1-15)	44.8	HUI 2	Initial Treatment<br>Surgery: 14<br>Limited surgery + radiotherapy: 15	 0.716<br>0.85	n/a	n/a
Wright et al.<br>2003<br>Canada	cross-sectional	survivors of ALL & disease-free controls	n/a	previously treated patients	ALL: 12.1 (3.6)<br>controls: 11.9 (2.9)	53.2<br>47.9	HUI 2	ALL: 62<br>Controls: 71	0.91 (0.084)<br>0.96 (0.057)	n/a	n/a
							HUI 3	ALL: 62<br>Controls: 71	0.86 (0.166)<br>0.96 (0.077)	n/a	n/a
Wright et al.<br>2005<br>Canada	cross-sectional	survivors of ALL	n/a	previously treated patients	survivors: 12.1 (4.9)<br>controls: 12.2 (5.2)	55.6<br>49.4	HUI 3	survivors: 77<br>controls: 71	0.86 (0.18)<br>0.97 (0.08)	n/a	n/a
Yaris et al.<br>2001<br>Turkey	Non-randomized, non-comparative, prospective;<br>3 months	newly diagnosed cancer	treatment for cancer	n/a	9.7 (4.6)	53.3	HUI 2	30	0.81 (0.12)	30	0.85 (0.11)
Sung et al.<br>2003<br>Canada	cross-sectional	inpatients undergoing chemotherapy & non-oncology outpatients (controls)	n/a	Inpatients & outpatients	inpatients: 7.2 (4.0)<br>controls: 10.1 (4.1)	64<br>43	HUI 2	inpatients: 36<br>controls: 49	0.85 (0.18)<br>0.80 (0.19)	n/a	n/a
							HUI 3	inpatients: 36<br>controls: 49	0.84 (0.23)<br>0.76 (0.24)	n/a	n/a
							SG	inpatients: 36<br>controls: 49	0.83 (0.29)<br>0.93 (0.12)	n/a	n/a
							TTO	inpatients: 36<br>controls: 49	0.64 (0.33)<br>0.88 (0.14)	n/a	n/a

SD-standard deviation; n/a-not available; CNS-central nervous system; ALL-acute lymphoblastic leukemia
