# Supplementary material for: A review of health utilities across conditions common in paediatric and adult populations
Source: Health Qual Life Outcomes. 2010 Jan 27;8:12. doi: 10.1186/1477-7525-8-12 (PMC2828427; doi:10.1186/1477-7525-8-12)
Supplement: Additional file 4 — Table S4 - Utilities derived for chronic disease. Table showing utilities derived for chronic disease, in PDF format. [file 1477-7525-8-12-S4.PDF]

Table S4 - Utilities derived for chronic disease

Author, Year Country	Study Design	Condition	Interven-tions	Setting	Mean (SD) Age	% Males	Utility Instrument	Baseline Utility		End of Study Utility	
								N	Mean (SD)	N	Mean (SD)
**Children/ Adolescents**											
Janse et al. 2005 Netherlands	cross-sectional	ALL, juvenile chronic arthritis, asthma, cystic fibrosis	n/a	tertiary pediatric centers	8.5 (4.2)	48	**HUI 3**	Parents: 279<br>Pediatricians: 279	0.80<br>0.93	n/a	n/a
Sung et al. 2004 Canada	cross-sectional	chronic disease	n/a	oncology ward & outpatient rheumatology, hemophilia, or bone marrow transplantation clinics	Patients: 13.7 (1.7)<br>Parents: 43.3 (4.7)	55<br>18	**HUI 2**	Patients: 22<br>Parents: 22	0.95 (0.07)<br>0.82 (0.22)	n/a	n/a
							**HUI 3**	Patients: 22<br>Parents: 22	0.92 (0.09)<br>0.79 (0.26)	n/a	n/a
							**TTO**	Patients: 22<br>Parents: 22	0.92 (0.11)<br>0.77 (0.31)	n/a	n/a

SD-standard deviation; n/a-not available; ALL-acute lymphoblastic leukemia
