# Supplementary material for: A review of health utilities across conditions common in paediatric and adult populations
Source: Health Qual Life Outcomes. 2010 Jan 27;8:12. doi: 10.1186/1477-7525-8-12 (PMC2828427; doi:10.1186/1477-7525-8-12)
Supplement: Additional file 5 — Table S5 - Utilities derived for type 1 diabetes mellitus. Table showing utilities derived for type 1 diabetes mellitus, in PDF format. [file 1477-7525-8-12-S5.PDF]

Table S5 - Utilities derived for type 1 diabetes mellitus

Author, Year, Country	Study Design	Interven-tions	Setting	Mean (SD) Age	% Males	Utility Instrument	Baseline Utility N	Baseline Utility Mean (SD)	End of Study Utility N	End of Study Utility Mean (SD)
**Children/ Adolescents**										
Nordfeldt et al. 2001 Sweden	Non-randomized, prospective cohort; 12 months	n/a	postal survey of previously treated patients (n=129)	11.8 (4.1)	n/a	**EQ-5D index**	n/a	Severe hypoglycemia median: 0.85<br>No severe hypoglycemia median: 1.0	n/a	n/a
**Adults**										
Currie et al. 2006a UK	Cross-sectional	n/a	postal survey of previously treated patients	54.8 (17.8)	51.7	**EQ-5D index**	236	0.57	n/a	n/a
Currie et al. 2006b UK	Cross-sectional	n/a	postal survey of previously treated patients	53.5 (18.2)	50.5	**EQ-5D index**	419	Effect of worst hypoglycemic event<br>None: 0.644 (0.34)<br>Mild: 0.758 (0.261)<br>Moderate: 0.68 (0.341)<br>Severe: 0.522 (0.422)	n/a	n/a
Hart et al. 2005 Netherlands	Non-randomized, prospective cohort followed for 6 years	none	outpatient clinic	38.2 (11.5)	57.3	**EQ-5D index**	234	1995: 0.90 (0.15)	230<br>220<br>217<br>213	1996: 0.88 (0.16)<br>1997: 0.87 (0.17)<br>1998: 0.87 (0.18)<br>2001: 0.85 (0.19)
Hart et al. 2003 Netherlands	Cross-sectional	n/a	outpatient clinic	38.2 (12.4)	54.4	**EQ-5D index**	274	0.88 (0.17)	n/a	n/a
Lee et al. 2005 UK	Cross-sectional	n/a	postal survey of previously treated patients	51.9	n.r	**EQ-5D index**	n/a	Type 1 DM with normal BMI: 0.706 (0.298)	n/a	n/a
Chancellor et al. 2008 UK	Cross-sectional patient preference study	n/a	community	49.2	64	**EQ-5D index**	132	0.75	n/a	n/a
						**TTO**	132	0.83 (0.02)	n/a	n/a
Supina et al. 2006 Canada	Cross-sectional	n/a	postal survey of previously treated patients	37.13 (14.3)	41.2	**HUI 3**	213	0.78 (0.23)	n/a	n/a
Kiberd & Larson 2000 USA & Canada	Cross-sectional; economic evaluation	n/a	community	n/a	62.5	**SG**	16	Insulin alone: 0.75 (0.05)<br>With pancreas transplant: 0.95 (0.02)<br>With pancreas-kidney transplant: 0.95 (0.02)<br>With kidney transplant: 0.72 (0.05)<br>With dialysis: 0.57 (0.05)<br>With blindness and kidney transplant: 0.49 (0.07)<br>With blindness: 0.48 (0.06)<br>With dialysis and blindness: 0.40 (0.06)	n/a	n/a
Brown et al. 2000 USA	Cross-sectional	n/a	hospital & physican practice (n=292)	61.7	44	**TTO**	Type 1: 72	0.88 (0.117)	n/a	n/a
Landy et al. 2002 USA	Cross-sectional	n/a	clinic, community	Type 1 DM: 61.8 (12.5)<br>Public: 44.6 (12.5)<br>Clinicians: 29.1 (7.3)	n.r	**TTO**	Type 1 DM: 85<br>General public: 157<br>Clinicians: 61	0.873<br>0.900<br>0.829	n/a	n/a

SD-standard deviation; n/a-not available; DM-diabetes mellitus
