# Supplementary material for: A review of health utilities across conditions common in paediatric and adult populations
Source: Health Qual Life Outcomes. 2010 Jan 27;8:12. doi: 10.1186/1477-7525-8-12 (PMC2828427; doi:10.1186/1477-7525-8-12)
Supplement: Additional file 6 — Table S6 - Utilities derived for skin disease. Table showing utilities derived for skin disease, in PDF format. [file 1477-7525-8-12-S6.PDF]

Table S6 - Utilities derived for skin disease

Author, Year, Country	Study Design	Condition	Interventions	Setting	Mean (SD) Age	% Males	Utility Instrument	Baseline Utility N	Baseline Utility Mean (SD)	End of Study Utility N	End of Study Utility Mean (SD)
**Children/Adolescents & Adults**											
Klassen et al. 2000 England	Non-randomized, prospective; 12 months	Acne	Isotretinoin or antibiotic, hormonal, physical or topical treatments	Dermatology clinic	22.1 (5.2)	61.3	**EQ-5D index**	54	0.84 (0.17)	54	0.93 (0.15)
Mittmann et al. 1999 Canada	Cross-sectional	Acne	n/a	National health survey (n=17,626)	n/a (age $\geq$12)	45.7	**HUI 3**	Overall: 476<br>Age 12-19: 85<br>Age 20-29: 48	0.92 (0.09)<br>0.92 (0.90)<br>0.92 (0.09)	n/a	n/a
**Children/Adolescents**											
Stevens et al. 2005 Germany	Cross-sectional; adults surveyed about children's preferences	Atopic dermatitis	n/a	General population survey	54	40.1	**SG**	137	0.841	n/a	n/a
Chen et al. 2008 USA	Cross-sectional	Acne	n/a	Four high schools	15.4 (1.3)	41	**TTO**	265	0.961 (0.092)	n/a	n/a
**Adult**											
Bergstrom et al. 2003 USA	RCT, 14 days	Psoriasis ≥ 3% of body surface area	Clobetasol propionate: foam vs combined cream & solution	Clinic	49	79	**EQ-5D index**	n/a	n/a	29	Foam: change -0.71<br>Cream/solution: change -0.13
Revicki et al. 2008 8 in Europe + Canada	RCT, 16 weeks	Moderate to severe plaque psoriasis	Adalimumab; MTX; placebo	28 centres	Placebo: 40.7 (11.4)<br>MTX: 41.9 (11.9)<br>Adalimumab: 42.8 (12.3)	66<br>67<br>64	**EQ-5D index**	53<br>108<br>103	Placebo: 0.7 (0.3)<br>MTX: 0.7 (0.2)<br>Adalimumab: 0.7 (0.3)	53<br>108<br>103	Placebo: 0.8 (0.3)<br>MTX: 0.9 (0.2)<br>Adalimumab: 0.9 (0.1)
Shikiar et al. 2007 USA & Canada	Phase II RCT, 12 weeks	Moderate to severe plaque psoriasis	Adalimumab (weekly or EOW) vs placebo	18 centres	Placebo: 43<br>Adalimumab EOW: 46<br>Adalimumab Weekly: 44	65<br>71<br>66	**EQ-5D index**	52<br>45<br>50	Placebo: 0.67<br>Adalimumab EOW: 0.69<br>Adalimumab Weekly: 0.69	52<br>45<br>50	Placebo: 0.68<br>Adalimumab EOW: 0.89<br>Adalimumab Weekly: 0.86
Shikiar et al. 2006 USA & Canada	RCT, 12 weeks	Moderate to severe plaque psoriasis	Adalimumab vs placebo	Multicentre	44.2 (12.7)	67.3	**EQ-5D index**	147	0.66 (0.28)	140	0.82 (0.23)
Stratton et al. 2001 UK	Non-randomized, prospective, pilot study, 12 months	Scleroderma	Anti-human lymphocyte globulin plus mycophenolate mofetil	Connective tissue diseases unit	Median: 52	26	**EQ-5D index**	13	0.54 (0.073)	13	0.49 (0.076)
Saarni et al. 2006 Finland	Cross-sectional	Psoriasis	n/a	General population survey (n=6681)	52.6	47	**EQ-5D index**	Psoriasis: 2.3% of population	0.796 (0.019)	n/a	n/a
Weiss et al. 2002 USA	Cross-sectional study of patients enrolled in RCT	Moderate to severe psoriasis	n/a	NIH clinical centre	49	60	**EQ-5D index**	35	0.724	n/a	n/a
Littenberg et al. 2003 USA	Longitudinal cohort test-retest reliability; 1-2 weeks	Stable skin disease	None	Postal questionnaire and dermatology clinic	51.9	24.3	**SG**	Overall: 74<br>Acne: 6<br>Keratosis: 16<br>Nevus: 6<br>Psoriasis: 5	0.9776<br>0.999<br>0.989<br>0.975<br>0.925	74	0.9779
Lundberg et al. 1999 Sweden	Cohort	Psoriasis; atopic eczema	None	Dermatology outpatient clinic	Psoriasis: 49.9 (13.1)<br>Eczema: 34.8 (12.0)	51<br>29	**SG**	Psoriasis only: 77<br>Psoriasis total: 234<br>Atopic eczema only: 34<br>Eczema total: 132	0.99 (0.044)<br>0.97 (0.107)<br>1.00 (0.102)<br>0.98 (0.069)	n/a	n/a
							**TTO**	Psoriasis only: 77<br>Psoriasis total: 234<br>Atopic eczema only: 34<br>Eczema total: 132	0.93 (0.132)<br>0.88 (0.153)<br>0.95 (0.128)<br>0.93 (0.115)	n/a	n/a
Khanna et al. 2007 USA	Cross-sectional	Systemic sclerosis	n/a	Conference attendees	54.4 (12.7)	9.4	**SG**	107	0.76 (0.28)	n/a	n/a
							**TTO**	107	0.76 (0.25)	n/a	n/a

Zug et al.<br>1995<br>USA	Cross-sectional	Psoriasis	n/a	Dermatology clinic	Median: 49	54	**SG**	Psoriasis: 82<br>covers <10% of skin<br>covers 10-30% of skin<br>covers >30% of skin	 0.82<br>0.78<br>0.62	n/a	n/a
							**TTO**	Psoriasis: 82<br>covers <10% of skin<br>covers 10-30% of skin<br>covers >30% of skin	 0.89<br>0.79<br>0.59	n/a	n/a
Chen et al.<br>2004<br>USA	Cross-sectional	Psoriasis;<br>dermatitis;<br>acne	n/a	Hospital (n=250)	46 (17)	43	**TTO**	Psoriasis: 11<br>Acne: 28<br>Atopic dermatitis: 5<br>Contact dermatitis: 10<br>Eczema and xerosis: 11<br>Lichen simplex chronicus: 5	0.907 (0.121)<br>0.938 (0.124)<br>0.890 (0.134)<br>0.898 (0.159)<br>0.968 (0.055)<br>0.987 (0.022)	n/a	n/a
Schiffner et al.<br>2002<br>Germany	Cross-sectional	Port wine stains on the face	n/a	Survey of patients who had completed laser therapy (n=25)	42	52	**TTO**	20	0.95	n/a	n/a
Schiffner et al.<br>2003<br>Germany	Pre-post design, prospective; 12 weeks	Psoriasis	Balneo-phototherapy	Physican	43.9	60.9	**TTO**	138	0.888	134	0.904
Schmitt et al.<br>2008<br>Germany	Cross-sectional	Psoriasis;<br>atopic eczema	n/a	Community	Psoriasis: 49.8<br>Eczema: 27.8	62.1<br>27.4	**TTO**	Psoriasis: 58<br>controlled<br>uncontrolled<br>Eczema: 62<br>controlled<br>uncontrolled	 0.88<br>0.45<br><br>0.96<br>0.65	n/a	n/a

SD-standard deviation; n/a-not available; RCT-randomized controlled trial
